# Supplementary material for: Analyzing COVID-19 disinformation on Twitter using the hashtags #scamdemic and #plandemic: Retrospective study
Source: PLoS One. 2022 Jun 22;17(6):e0268409. doi: 10.1371/journal.pone.0268409 (PMC9216575; doi:10.1371/journal.pone.0268409)
Supplement: S1 File — (DOCX) [file pone.0268409.s001.docx]

**Supplementary Information for:**

Analyzing COVID-19 Disinformation on Twitter using the Hashtags #scamdemic and #plandemic

**Authors:**

Heather D. Lanier, MPH

Marlon I. Diaz

Sameh N. Saleh, MD

Christoph U. Lehmann, MD

Richard J. Medford, MD

**Corresponding Author:**

Heather D. Lanier, MPH

[Heather.lanier@utsouthwestern.edu](mailto:Heather.lanier@utsouthwestern.edu)

Section A. Methods: data processing, transformation, and exploration.

We analyzed the metadata we obtained from the tweets to provide descriptive characteristics such as sentiment analysis, topic modeling, and emotion analysis. To do this, we used several natural language processing techniques to transform the tweets into plain text, which required the removal of hyperlinks, user mentions, user replies, and the removing the “#” symbol from the hashtags. We furthered transformed the plain text tweets by removing stop words (which are frequently used words the provide little to no semantic meaning words such as “it”, “the”, and “as”)^[1]^. For the sentiment analysis, we used the SentimentIntensityAnalyzer module of the VADERSentiment library in Python^[2]^ using the plain text tweets. We then took the average weekly sentiment of all the tweets, separated by hashtag, starting the first week of April, 2020.

**Table S1.** Metadata associated with tweets

| user_id | media_url | profile_url |
| --- | --- | --- |
| status_id | media_type | profile_background_url |
| created_at | ext_media_url | account_created_at |
| screen_name | ext_media_type | verified |
| source | user_mentions | profile_image_url |
| reply_to_status_id | quoted_text | name |
| is_quote | retweet_status_id | location |
| is_retweet | place_name | description |
| favorite_count | protected | account_lang |
| retweet_count | followers_count | profile_banner_url |
| reply_count | friends_count | ang |
| hashtags | statuses_count |  |
| urls_url | favourites_count |  |

**Figure S1.** Reporting misleading information on Twitter


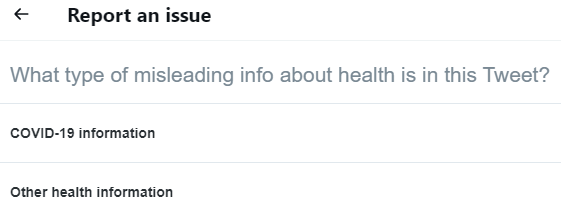


**References:**

1. Loper E, Bird S. NLTK: the Natural Language Toolkit. In: Proceedings of the ACL-02 Workshop on Effective tools and methodologies for teaching natural language processing and computational linguistics - [Internet]. Philadelphia, Pennsylvania: Association for Computational Linguistics; 2002 [cited 2020 Jul 6]. p. 63–70. Available from: <http://portal.acm.org/citation.cfm?doid=1118108.1118117>
2. Hutto, C.J. & Gilbert, E.E. (2014). VADER: A Parsimonious Rule-based Model for Sentiment Analysis of Social Media Text. Eighth International Conference on Weblogs and Social Media (ICWSM-14). Ann Arbor, MI, June 2014.
