# Supplementary material for: Analyzing COVID-19 disinformation on Twitter using the hashtags #scamdemic and #plandemic: Retrospective study
Source: PLoS One. 2022 Jun 22;17(6):e0268409. doi: 10.1371/journal.pone.0268409 (PMC9216575; doi:10.1371/journal.pone.0268409)
Supplement: Attachment — Submitted filename: Responses to Reviewer Comments.docx [file pone.0268409.s003.docx]

| Reviewer Comment | Our Response |
| --- | --- |
| The literature review should be precisely and concisely presented. The gap of knowledge and necessity of the study should be precisely stated. | We appreciate this sentiment and hope that we improved significantly here. The introduction now contains “The hashtags #scamdemic and #plandemic, which imply that the pandemic is a conspiracy, are frequently associated with intentional disinformation, however tweets with these hashtags have not been examined to explore the scope of disinformation. Understanding the extent and impact of false information is important for officials and public health agencies to predict population behavior including the potential uptake of vaccines and non-pharmaceutical measures such as masking and social distancing. Our hypothesis was that analysis of tweets associated with these hashtags would provide valuable insight about disinformation and the public’s beliefs around the COVID-19 pandemic and would aid in developing targeted public health interventions.” |
|  |  |
| Coherence within paragraph and between paragraphs and sentence sequence were not well designed | We appreciate the comment and hope that we were able to improve on transitions. |
| What is statement “Error! Bookmark not defined” in line78 | Thank you for this comment. This error has been resolved and should no longer be seen in the submission. |
| The statistical analysis should be precisely stated. | Thank you for this comment. Statistical tests that were used are included at the bottom of each table in the footnotes. We agree that this addition is important. |
| Time of data collection should be clarified and be the same in all parts of manuscript. | Thank you for this comment. After reviewing, we believe that our manuscript accurately describes the time of data collection, with January 3^rd^ being the date of the initial scraping and January 15^th^ being the date that we used the Twitter API to extract additional relevant metadata to the associated tweets. Through this extraction on January 15^th^, we also determined the status of tweets and users. |
| Some result of data collection mention in method section l96-98. Please move to result section. | Thank you for this comment. This result has been moved from methods to results section. |
| Some percentage in the result’s text and tables were over 100%. please revise them. | Thank you for this comment. These percentages have been revised. |
| It is not necessary to bring all the table’s contents in the text, just mention main results. | We appreciate the comment but were unable to identify the table in question. We suspect that the comment refers to table 1 and we modified the result section to “The largest group of users were 40 years or older. Males and Non-Hispanic Whites represented the largest groups. (Table 1) Male users and users in the age groups <= 18 years and 30-39 years were overrepresented significantly among the suspended users. The vast majority of active and suspended users tweeted from personal accounts, 88.2% and 79.4% respectively.” |
| Discussion is weak and repeats the results, please rewrite again or improve them. | We abstracted more and we added substantially to the discussion especially the section on suspended users to put our findings in context. |
| 1. In your emotion analysis, the authors label the ten emotions for each tweet: fear, anger, anticipation, trust, surprise, positive, negative, sadness, disgust, or joy. These are not common basic emotions. Is there theoretical support for the choice of these emotions? | Thank you for this comment. The NRCLex library contains a larger dataset compared to other emotional analysis tools available. It has also been used in several other publications.  [NRC Emotion Lexicon (saifmohammad.com)](https://saifmohammad.com/WebPages/NRC-Emotion-Lexicon.htm) |
| 2. At the beginning of the discussion, the authors describe different types of false information in social media. Isn’t it better to be described in the introduction? | Thank you for this comment. The introduction has been modified to include this explanation of different types of false information in social media. |
| 3. The authors mention this paper can aid in developing targeted public health interventions. Could the authors write down several paragraphs about what kind of interventions can be developed to tackle the disinformation about the COVID-19 pandemic based on the findings? Adding this implication part in the discussion may add more value to your paper. | We appreciate this comment but we were reluctant as these interventions were not part of the paper. However, we added “Our study demonstrates that it is possible to identify disinformation from tweets. In the future, public health agencies could automate the tools used to identify disinformation in real time and target it with replies that disseminate correct but related educational information. We envision public health “bots” as a means of de-arming disinformation spreaders.” |
| Useful research, it would be nice to extend the conclusions with some results' implications part - with one targeting social implications - educational ones. Even without this implications part, conclusions might be more elaborated, they have been synthesized to a minimum. | Again, we are concerned to stray too far from the results in the discussion. We added the sentence on educational bots as mentioned above. |
